# PPARs are a unique set of fatty acid regulated transcription factors controlling both lipid metabolism and inflammation^☆^

**DOI:** 10.1016/j.bbadis.2011.02.014

**Published:** 2011-08

**Authors:** Tamas Varga, Zsolt Czimmerer, Laszlo Nagy

**Affiliations:** a Department of Biochemistry and Molecular Biology, Research Center for Molecular Medicine, University of Debrecen, Medical and Health Science Center, Hungary; b Apoptosis and Genomics Research Group of the Hungarian Academy of Sciences at the University of Debrecen, Medical and Health Science Center, Hungary

**Keywords:** PPAR, Inflammation, Lipid metabolism, Transcriptional regulation

## Abstract

Cells are constantly exposed to a large variety of lipids. Traditionally, these molecules were thought to serve as simple energy storing molecules. More recently it has been realized that they can also initiate and regulate signaling events that will decisively influence development, cellular differentiation, metabolism and related functions through the regulation of gene expression. Multicellular organisms dedicate a large family of nuclear receptors to these tasks. These proteins combine the defining features of both transcription factors and receptor molecules, and therefore have the unique ability of being able to bind lipid signaling molecules and transduce the appropriate signals derived from lipid environment to the level of gene expression. Intriguingly, the members of a subfamily of the nuclear receptors, the peroxisome proliferator-activated receptors (PPARs) are able to sense and interpret fatty acid signals derived from dietary lipids, pathogenic lipoproteins or essential fatty acid metabolites. Not surprisingly, Peroxisome proliferator-activated receptors were found to be key regulators of lipid and carbohydrate metabolism. Unexpectedly, later studies revealed that Peroxisome proliferator-activated receptors are also able to modulate inflammatory responses. Here we summarize our understanding on how these transcription factors/receptors connect lipid metabolism to inflammation and some of the novel regulatory mechanisms by which they contribute to homeostasis and certain pathological conditions. This article is part of a Special Issue entitled: Translating nuclear receptors from health to disease.

## Research highlights

► PPARs and lipid environment form a regulatory circuit. ► Fatty acid derived molecules can regulate inflammation through PPARs. ► PPARs have anti-inflammatory roles in a number of inflammatory diseases. ► A number of outstanding questions about the biology of PPARs are still unanswered.

## 1 Introduction

Early in the '90s it was observed that several distinct compounds with similar chemical properties were able to trigger both an increase in size and number of hepatic and renal peroxisomes in rodent cells. Treatment of rodents with these compounds also increased the rate of β-oxidation of fatty acids and caused hepatomegaly and carcinogenesis, which did not occur in humans [1]. These compounds included certain herbicides, phthalate plasticizers and, most importantly, the fibrate class of hypolipidemic drugs [2,3], which were later termed as peroxisome proliferators. The search for the pharmacophores whose activation by peroxisome proliferators caused the above effects led to the identification of peroxisome proliferator-activated receptor alpha (PPARα), a novel member of the nuclear receptor hormone superfamily [1]. Based on sequence homology, the genes for two other members of the PPAR subfamily, PPARγ and PPARβ/δ, were later cloned from the mouse genome [4–6]. All three subtypes of the PPAR subfamily were found to be highly expressed in tissues relevant to energy homeostasis, and since certain dietary fatty acids and their metabolic derivatives were found to activate PPARs, the idea was instantly formulated that PPARs were regulators of metabolism. The validity of this suggestion was thoroughly proven in human and mouse studies revealing that PPARs are indeed master regulators of metabolism [7]. Later, better understanding of the expression pattern, activity and biology of these transcription factors indicated that they also have diverse functions outside of the realm of metabolism. Not surprisingly, among these non-canonical PPAR functions the regulation of inflammation has received the most attention and has been the focus of intense research effort. Numerous studies indicate that PPARs have anti-inflammatory effects in a wide range of pathological conditions. This review aims to summarize both the fundamental findings of PPAR biology and the advances in the understanding of PPARs' functions in inflammatory responses.

## 2 Lipid biology and inflammation

Inflammation is a multistep process by which the host responds to the perturbation of homeostasis. Microbial infections, injuries or altered physiological conditions all have the potential to trigger an inflammatory reaction. Upon elimination of the source of inflammation, a resolution phase ensues in which the damaged surrounding tissue is repaired and homeostasis is restored. Inflammatory reactions can be divided into acute inflammation, which is resolved in a relatively short time, hours or days, and chronic inflammation, which occurs over a longer period of time. Typically, chronic inflammatory reactions develop gradually from an acute reaction if the host is not able to eliminate the inflammatory inducer (for a review of inflammation, see [8]). There is another form of chronic inflammatory response, which is not linked to an acute event. Altered homeostatic conditions, such as obesity and the related type 2 diabetes, or atherosclerosis, exhibit certain features of an ongoing low grade systemic chronic inflammation, including elevated levels of inflammatory cytokines or acute phase proteins produced by the liver.

An inflammatory reaction can be regulated in a number of different ways. The detection and interpretation of inflammatory signals, the response of the activated immune cells (such as cytokine release), the reaction of the surrounding tissue to inflammatory agents and to immune mediators as well as the resolution/repair phase are all targets of regulatory mechanisms. Interestingly, several regulatory mechanisms of the inflammatory reaction were demonstrated to be influenced by lipid molecules. Due to the fact that PPARs are specialized receptors to detect fatty acid derived signal molecules, they are key candidate for being the receptors that transduce a fraction of the lipid mediated inflammatory signaling events. Indeed, several instances were identified in which certain fatty acid derived molecules were shown to activate PPARs and modulate inflammation [9,10]. The best example of this might be the case of eicosanoids. It has been known for a long time, that eicosanoids, the products of the essential fatty acid metabolism, are potent inflammatory agents that act locally to modulate inflammation. The simplified picture suggests that eicosanoids are pro-inflammatory mediators that act locally to enhance vasodilatation and increased permeability of venules. However, a few examples of anti-inflammatory eicosanoids, such as lipoxins, have also been described (for a review see [11]). Additionally, it was found that a gradual shift in the eicosanoid profile of inflammatory reactions resulted in diminished production of the initially predominant pro-inflammatory leukotrienes and increased release of pro-resolution lipoxins in the course of the inflammatory reaction [12]. Importantly, certain eicosanoids can signal not only *via* their own cell surface receptors, but also *via* PPARs, suggesting that the regulatory effects of these eicosanoids are partly mediated by PPARs. Recently, emerging evidence has suggested that another class of fatty acid molecules may also have dual potential to activate PPARs and modulate inflammation. Dietary fatty acids have been regarded solely as energy source for a long time, but they are now also recognized as regulators of inflammation (often *via* the modulation of eicosanoid synthesis). For instance, saturated fatty acids and different classes of polyunsaturated fatty acids (PUFAs) were demonstrated to modulate inflammation [13]. Again, it is highly probable that PPARs play major roles in transducing the signals derived from dietary intake of lipids to the level of immune regulation.

The role of PPARs in inflammation is especially relevant in the case of metabolic syndrome and atherosclerosis. These are diseases of lipid (and glucose) metabolism with an underlying inflammatory component. Metabolic syndrome is a cluster of symptoms (impaired glucose tolerance, high blood pressure, dyslipidemia and abdominal obesity) that are often associated and significantly increase cardiovascular risk. Currently two main competing theories are proposed that could explain the emergence of impaired glucose tolerance in these patients. The lipotoxicity theory [14] proposes that when the fat storing capacity of the adipose tissue is chronically overloaded, fatty molecules will be deposited in other tissues, including muscle and liver. This would cause impaired insulin signaling in these tissues and, as a result, would lead to impaired glucose tolerance. The alternative explanation suggests that chronic caloric overload initiates an inflammatory response that is originated in the adipose tissue [15,16]. The inflammatory reaction make adipocytes and immune cells residing in the adipose tissue produce cytokines and adipokines (such as tumor necrosis alpha (TNFα)) that could lead to impaired glucose tolerance in remote tissues of the body [17]. Regardless of the nature of the primary mechanism, chronic inflammation seems to be an important component of metabolic disease. Synthetic PPARγ agonists (the glucose sensitizing thiazolidinedione (TZD) drugs) are potent drugs that improve insulin sensitivity in metabolic syndrome [18]. These drugs act primarily by modulating lipid and glucose metabolism, but they also have well documented anti-inflammatory effects as well. It is possible that PPARγ ameliorates metabolic syndrome solely by improving metabolic activities of target tissues involved in carbohydrate and lipid metabolism, as well as fat storage. It is also possible, however, that the beneficial effects of PPARγ activation on insulin sensitivity are mediated, at least partly, by its anti-inflammatory activities.

**Fig. 1:**
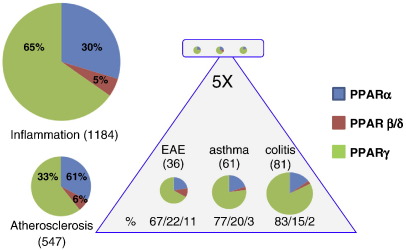
Number of publications on the role of PPARs in different types of inflammation. PubMed search was carried out with the combinations of the names of PPAR subtypes and certain inflammatory conditions. The total area of the pie diagrams correlates with the number of publications found (without reviews). Total number of publications (in parentheses) and percentage distribution of the three PPAR subtypes are indicated. The size of the sectors indicates the number of publications on distinct subtypes in inflammatory conditions.

Due to their role in the above medical conditions, the metabolic roles of PPARs have been the target of intensive research. As their functions in inflammation slowly emerged, an increasing number of studies were devoted to dissect the role of PPARs in different types of inflammation, as well (Fig. 1).

## 3 General biology of nuclear receptors

As already noted, PPARs belong to the nuclear receptor hormone superfamily. These proteins are transcription factors that are not only able to bind to DNA and regulate gene expression but they also serve as intracellular receptors by binding lipid molecules. This superfamily emerged in the early metazoan evolution and underwent an intensive evolutionary divergence [19,20]. As a result, the human and the mouse genome contain genes for 48 or 49 different nuclear receptors, respectively. A series of gene duplication events during early vertebrate evolution produced (among other subfamilies) the three members of the PPAR subfamily. There are several ways to group and categorize the divergent members of the superfamily. Originally those nuclear receptors whose ligands were identified were called “classic” nuclear receptors. These are typically endocrine receptors that bind their ligands with high affinity (such as thyroid hormone receptors for the thyroid hormones or the estrogen receptors for estrogens). Other nuclear receptors, whose ligands remained unknown, were classified as orphan nuclear receptors. To compound the naming system, the cognate ligands of some orphan nuclear receptors (including PPARs) were originally unknown but were later identified. Upon the discovery of their cognate ligands this later group of orphan nuclear receptors became “adopted” and was often referred to as adopted nuclear receptors [21]. This classical grouping system of nuclear receptors has several weaknesses. Maybe the biggest source of inconsistency in this classification derives from the fact that those nuclear receptors are categorized as orphans whose ligand has not been found yet. Since novel ligands are found and characterized all the time, this classification is, by its very nature, always temporary. Reflecting on the weaknesses of the above classification principle based on the presence of endogenous ligands, a phylogenetic classification model was introduced [22] in which the main organizing principle was sequence similarity. In this new nomenclature nuclear receptors received novel acronyms that reflected their position in the phylogenetic tree. Accordingly, PPARα, PPARβ/δ and PPARγ were renamed as NR1C1, NR1C2 and NR1C3, respectively. Although it would be more accurate to refer to PPARs according to this new naming system in the literature (including this review), we will use their trivial names to avoid confusion. One can assume that nuclear receptors that are very close on the phylogenetic tree and are therefore highly related show similar ligand binding properties and fulfill similar cellular roles. As their novel nomenclature indicates, the three different subtypes of PPAR show relatively high similarity. It would be tempting to assume therefore that they have similar functions. However, this is certainly not true for their main action, namely the regulation of metabolism, in which PPARα, an activator of mitochondrial and peroxisomal fatty acid β-oxidation in liver, PPARβ/δ, a regulator of fatty acid oxidation in muscle and PPARγ, an activator of fatty acid synthesis and storage, play divergent roles in metabolism. Surprisingly, the emerging body of studies on the inflammatory roles of the three PPAR subtypes suggests that all the three PPARs have, for the most part, anti-inflammatory activities. How the three PPAR subtypes, with divergent metabolic roles and distinct transcriptional activities, can have highly overlapping anti-inflammatory profiles is an open question.

## 4 Introduction to PPARs

As noted earlier, there are three subtypes of PPARs in vertebrates, PPARα (NR1C1), PPARβ/δ (NR1C2) and PPARγ (NR1C3). Mouse PPARα was cloned first, which was followed by the cloning of all three subtypes from *Xenopus* and other vertebrate genomes [4–6,23–26]. The naming of the mammalian homologues of PPARα and PPARγ was evident because they showed high degree of similarity to their counterparts found in the *Xenopus*. Mammalian PPARβ/δ, however, showed considerable divergence at the sequence level from the *Xenopus* PPARβ. Because of the lesser degree of sequence homology, this subtype in the mammals was alternatively named as PPARβ, PPARδ, FAAR, NUC1 or PPARβ/δ.

### 4.1 Ligands of PPARs

PPARs were originally described as orphan nuclear receptors, but soon a plethora of potential endogenous ligands were described. There are two major class of assays used to identify ligands. One class is represented by *in vitro* transactivation assays. In these cellular assays nuclear receptors are expressed ectopically in cells and the candidate ligands are used to activate the receptors. A marker of the transactivation activity of the nuclear receptor is then measured. This marker could be *e.g.* a known target gene for the corresponding receptor, or a signal that derives from an expression construct where a PPAR target sequence linked to a core promoter and a reporter (*e.g.* luciferase) gene. The second class of methods comprises biophysical techniques that measure the physical binding of candidate ligands to the receptors (*e.g.* radioligand competition assays, the Scintillation Proximity Assay (SPA), the Ligand Induced Complex (LIC) assay or the Coactivator Dependent Receptor Ligand Assay (CARLA)). The detailed description of these methods is outside the scope of this review. However, it is important to note that both classes of techniques carry their own weaknesses. When a candidate ligand causes enhanced gene expression in transactivation assays it is difficult to determine whether the candidate ligand itself binds to the receptor. Alternative explanations for the enhanced transactivation activity can be that the candidate ligand is actually a precursor of a true ligand, or that it initiates signaling events that will culminate in generating an unidentified ligand. The weakness of the biophysical methods is that although they provide a direct proof for the binding of a candidate ligand to the receptor, they do not guarantee that these molecules are present in the cells at high enough concentrations to serve as effective endogenous ligands. Indeed, most of the putative ligands are required to be present *in vivo* at such a high concentrations to efficiently activate PPARs that their function as *bona fide* PPAR ligands is often questioned. This leads to the paradox situation that although numerous ligands are suspected to be PPAR activators, the real identity of true endogenous ligands of PPARs is still very much debated. Here we provide a short summary of the major groups of the candidate PPARs ligands. More detail regarding the ligands of different PPAR subtypes will be provided in the corresponding sections.

Due to the fact that three subtypes do not show strict ligand specificity, it is logical to discuss the ligands of all PPAR subtypes together (Table 1). If a certain ligand is able to activate one subtype, then the same molecule is often found to bind to or activate the other subtypes at various efficiencies. Therefore a key feature of PPARs appears to be that they act as receptors that have numerous ligands that each binds to the receptors with relatively low affinity. This is in stark contrast with the case of the classic nuclear receptors (*e.g.* estrogen receptor or glucocorticoid receptor) that bind a very limited number of highly specific ligands at high affinity. This promiscuous ligand binding of PPARs is also reflected in the size of the ligand binding pocket of the PPAR proteins. The ligand binding pocket of PPARs is characteristically larger than that of classic nuclear receptors. It is possible that the unusually large ligand binding pocket enables PPARs to bind such a variety of different ligands.

Molecules that were found to bind physically to PPARs include polyunsaturated fatty acids (PUFAs) such as certain ω3-polyunsaturated fatty acids (*e.g.* α-linolenic acid with C18:3, or docosahexaenoic acid with C22:6), and certain ω6-polyunsaturated fatty acids (such as linoleic acid with C18:2 and arachidonic acid with C20:4). Certain saturated fatty acids (such as myristic acid with C14:0 and stearic acid with C18:0) were also found to bind to PPARα. The finding that dietary fatty acids can bind to and activate nuclear receptors and regulate gene expression akin to small molecule endocrine hormones caused a paradigm shift in how we think about the role of dietary fatty acids. Another group of PPAR ligands comprises the conversion products of essential fatty acids (mainly arachidonic acid) by lipoxygenases or cyclooxygenases. The best examples for eicosanoids that are possibly PPARα ligands are hydroxyeicosatetraenoic acids (*e.g.* 8(S)-HETE) and leukotriene B4 (LTB4), while prostacyclin (PGI2) [27], an eicosanoid with platelet activation inhibiting activity, is a possible endogenous ligand for PPARβ/δ. Other essential fatty acid metabolites, such as 15-deoxy-Δ12,14-prostaglandinJ2 (15d-PGJ2) and components of oxidized low-density lipoproteins, hydroxyoctadenoic acids (9-HODE and 13-HODE) are suspected PPARγ ligands [27–32]. Recent findings raised the interesting idea if PPARs are not specific receptors for one particular fatty acid molecule but are sensor molecules that sample the intracellular mixture of available fatty acid species. In line of this idea, Itoh et al. raised the possibility that PPARγ covalently binds a subset of fatty acids and that PPARγ can bind two ligand molecules at the same time [33]. The involvement of PPARs in detecting the fatty acid milieu was further supported in a study that demonstrated that PPARβ/δ is a hepatic free fatty acid sensor in the mouse [34].

Which of the above mentioned candidate ligands are *bona fide* endogenous ligands? To answer this question it would be necessary to know which candidate molecules are present intracellularly at a concentration that is in good agreement with their dissociation constant (Kd) values. Not only is this a technically extremely challenging task, but it is not fully understood how these lypophilic molecules are transported across the cell membrane, stored or metabolized in the cells. It is possible that certain candidate ligands that are present at low extracellular concentrations (which would seemingly preclude them from being endogenous ligands) can reach high concentrations locally. Among the candidate ligands listed above, the polyunsaturated fatty acids that can bind to PPARs are potentially true endogenous ligands for PPARα. It is less certain if any of the saturated fatty acids can work as ligands for PPARs. The eicosanoids (such as 8(S)-HETE for PPARα, 15d-PGJ2 for PPARγ and 15-HETE for PPARβ/δ) are often cited as lipid metabolites that have the potential to reach high enough concentrations to activate PPARs [35]. In summary, despite the large number of candidate endogenous ligands the true identity of the *bona fide* ligand(s) for PPARs is highly controversial.

PPARs also have synthetic ligands that can easily be used to interrogate the transcriptional activities of the PPARs in cells that express different subtypes of PPARs. All three subtypes have highly (but not exclusively) specific agonists [18,27,36–38]. Good examples are the hypolipidemic drugs clofibrate and fenofibrate, the potent synthetic ligand Wy-14643 for PPARα, the thiazolidinedione (TZD) group of antidiabetic drugs (including troglitazone, pioglitazone, ciglitazone and rosiglitazone (formerly known as BRL 49653)) for PPARγ and GW-501516 for PPARβ/δ. PPARγ also has a specific synthetic antagonist, called GW-9662. Certain nonsteroidal anti-inflammatory drugs (NAIDS) were also shown to activate PPARγ [39]. These drugs (including indomethacin, ibuprofen and fenoprofen) are cyclooxygenase inhibitors, but they have certain effects that could not be ascribed to inhibition of cyclooxygenases.

### 4.2 DNA recognition by PPARs

PPARs form heterodimers with their obligate partners, the members of another subfamily of nuclear receptors, RXRs (of which three subtypes exist, RXRα, RXRβ and RXRγ). According to the simplified model, upon ligand binding PPAR/RXR heterodimers recognize and bind to specific DNA sequences, called PPAR response elements (PPRE) [40]. This PPRE is a direct repeat of six nucleotide long core recognition motives (AGGTCA) that are separated by a single nucleotide. Because of the orientation and distance of the two hexameric motives, the PPRE is also called DR1. There are some additional features of PPAR target sites, such as the presence of an extended 5-half site, the presence of adenine as the separator single nucleotide or a slightly imperfect hexameric motif [41]. It is important to note, however, that the exact features of the consensus PPREs were calculated from DNA sequences to which PPARs were shown to bind and on which they acted as transactivators. The consensus sequence was derived from the 5′ region of known PPAR target gene transcriptional start sites that showed robust regulation by PPARs. Due to the fact that PPARs (and other nuclear receptors) can bind to DNA in both orientation and at an unpredictable distance to their target genes, it is possible that the fine features of the DR1 elements were calculated from sequences that were not perfectly representative to all PPREs. Novel experimental approaches, such as “ChIP-on-chip” or “ChIP-seq” that are based on determining the sequence of the DNA binding sites of chromatin immune-precipitated transcription factors hold the potential to identify and fully characterize PPREs at the whole genome level [42,43].

All three PPAR subtypes are believed to bind to canonical DR1 elements. Due to the fact that certain cell types express more than one PPAR subtype, the question arises what determines PPAR binding to a certain DR1 element in these cells [44]. Results showed that the 5′ flanking nucleotides of the core DR1 elements played an important role in determining the PPAR subtype specificity of PPREs. Still, these fine additional sequence features cannot make PPREs entirely subtype specific. Accordingly, there are only a very few, if any, PPAR responsive genes that can only be regulated by one subtype of PPARs. A recent example of a subtype specific response element was shown to be present in the fatty acid binding protein 4 (FABP4/aP2) gene [45] that is under the exclusive control of PPARγ in macrophages but not in adipocytes.

A further twist in the recognition of DR1 elements by PPAR-RXR heterodimers is the fact that the heterodimeric partner, RXR, is also a nuclear receptor and has its own cognate ligand (*e.g.* 9-cis retinoic acid or various fatty acids). It is possible that in cells in which there is no PPAR ligand available but RXR ligands are present, functional PPAR-RXR heterodimers can still bind to DR1 elements and regulate gene expression. When and to what extent this might happen is a complex question, and could be influenced by the target gene, the cell type and possibly by uncharacterized fine features of DR1 elements [46].

### 4.3 Experimental models for studying PPAR functions

One of the reasons why PPARs have been studied very intensively is that synthetic ligands of PPARα and PPARγ have been commonly used to treat metabolic diseases, such as dyslipidemia and type 2 diabetes, respectively, that affect millions of patients worldwide. As a result, the early studies focused on the metabolic actions of PPARs. Concomitant to the experimental studies, enormous amount of data accumulated over the years about the long term medical effects of the pharmacological activation of PPARs. This wealth of information can give us a head start in understanding the functions of PPARs in fields not directly related to metabolism.

There are several types of experimental approaches from which we have learned a great deal about the inflammatory roles of PPARs. The simplest approach is to analyze transcriptional events in cells in which PPARs are activated by ligand treatment. Synthetic agonists are available for each PPAR subtypes. Additionally, a very specific antagonist for PPARγ also exists. The availability of these compounds gives us a unique opportunity to study these transcription factors. The fact that agonists for PPARα and PPARγ were characterized first partly explains why we know much more about the functions of these PPARs. It must be noted, however, that results based on transcriptional profiling of agonist treated cells can be misleading. Although PPAR ligands are highly (subtype) specific for the most part, it is also well documented that there are agonist effects that are mediated by the other PPARs or not mediated by PPARs at all, especially when these agonist are applied at very high concentrations.

PPAR functions can also be studied in animal models of disease. There are genetically modified mouse strains available for each PPAR. Whole body knockout of PPARα causes relatively minor phenotypes in unchallenged animals [47,48]. PPARβ/δ knockout animals display high embryonic mortality on inbred background [49]. PPARγ^−/−^ animals are not viable and die *in utero* due to the deleterious effects of PPARγ deficiency in placentation. As a corollary, deficiency in PPARγ (and partly in PPARβ/δ) can be studied in heterozygous animals or in mice in which the PPARγ gene is disrupted by a Cre/lox mediated deletion only in certain cell types [50,51]. These animals, in which PPARγ is deleted only in *e.g.* macrophages, T cells or adipocytes, are viable and breed easily, but often show increased susceptibility to different experimentally induced diseases. A recently applied strategy used to circumvent the deleterious effects of placental PPARγ deficiency makes it possible to generate full body PPARγ knockout animals in which PPARγ functions are preserved in placental trophoblasts but lost in virtually all embryonic tissues. In these mouse models the Cre recombinase was under the control of the Mox or the Sox2 promoter [52,53]. The latter model appears to be superior due to a more throughout recombination in the embryo proper. The role of PPARs in various diseases can also be studied by treating wild type animals, in which distinct diseases are developed experimentally, with PPAR ligands. Again, agonists were found to have considerable off-target effects in the animal studies. As a result, effects of agonist treatments in wild type animals can only be ascribed to PPAR mediated functions if these effects are not detectable in PPAR deficient animals.

Finally, clinical studies, medical practice and human genetics are also important sources of information that can help dissect the functions of PPARs. Millions of people worldwide have been treated with synthetic agonists of PPARγ for impaired glucose tolerance, or with PPARα agonists to normalize serum lipid levels. Statistical analysis of the incidence of diseases where PPARs are implicated can corroborate or refute results derived from cellular or animal studies.

### 4.4 Mechanism of the inflammatory actions of PPARs

**Fig. 2:**
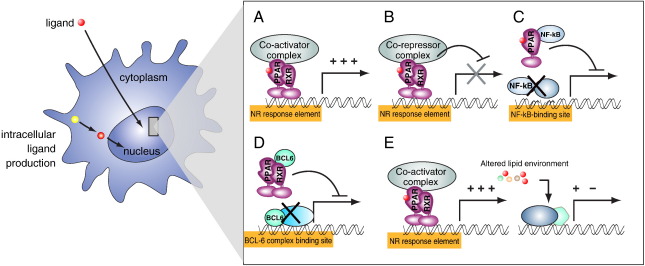
Mechanisms of genetic regulation by PPARs. (A) Upon ligand binding, PPARs induce gene expression. A subset of the induction, shown here, is the result of the direct regulation of gene expression by transactivation. Liganded PPAR/RXR heterodimers recruit co-activator molecules to promoters that contain PPAR response elements and subsequently activate gene expression. (B) A subset of direct target genes might be repressed by PPARs in the presence of ligands. However, the majority of characterized PPAR-mediated transcriptional regulations result in activation. (C) Ligand dependent *trans*-repression by PPARs. Upon ligand binding, PPARs can interfere with the activity of distinct transcription factors, such as NF-κB, through protein–protein interactions. (D) Ligand independent transrepression. Unliganded PPARs can bind and sequester transcription factors blocking their activity. A typical example for such a mechanism is the binding of BCL-6 by unliganded PPARβ/δ. (E) PPARs alter systemic lipid homeostasis which can affect gene regulation through unrelated transcription factors.

Cellular and *in vivo* studies suggest that PPARs can exert their anti-inflammatory effects by several distinct molecular mechanisms (Fig. 2). As transcription factors, their canonical regulatory mechanism is transactivation, by which ligand activated PPARs bind to their recognition sequences and regulate gene expression. Another form of gene regulation by PPARs is transrepression. During transrepression, PPARs bind and sequester other, unrelated transcription factors or transcriptional regulators. As a net result of this sequestration, regulatory circuits regulated by the relevant transcription factors will be impaired. Such transrepression mechanisms have been described for each subtype of PPARs. PPARα and PPARγ were described to transrepress other transcription factors when they are activated by ligand binding. On the other hand, most of the known anti-inflammatory effects of PPARβ/δ are mediated by ligand independent transrepression.

How are the molecular mechanisms of PPAR activity translated into anti-inflammatory effects? It is possible that PPARs regulate the expression of genes (either directly or indirectly, through transrepression) that have direct inflammatory roles. Alternatively, it is also possible that PPARs modulate inflammatory processes indirectly by altering lipid metabolism. According to this scenario, PPARs would directly modify the intra-, and extracellular pool of lipid molecules available in the body, and this altered lipid environment would initiate secondary regulatory processes. Such a mechanism is described in human dendritic cells (DCs) [54] where activation of PPARγ leads to the generation of retinoic acid, a molecule that regulates DC phenotype. It must be noted that the three PPAR subtypes alter intra-, and extracellular lipid homeostasis in distinct ways. PPARα is activated and provides energy from fatty acid catabolism during starvation and cold acclimatization, PPARγ is activated in the well fed state and regulates the synthesis of fatty acids and related lipids, while PPARβ/δ ensures, among other, that fatty acids can provide energy for working muscles. How the three subtypes of PPAR that alter lipid metabolism in three distinctly different ways and generate distinct classes of lipid molecules can have similar anti-inflammatory roles in diseases suggest that the regulatory circuits of PPARs in metabolism and inflammation are, at least partly, uncoupled. It is also probable that the observed anti-inflammatory effects of PPARs are not exclusively mediated through their capacity to alter whole body lipid homeostasis and direct molecular regulatory mechanisms are, at least partly, accountable for their anti-inflammatory effects. In the next sections we provide a short summary of the biology of the different subtypes and present a subjective account of the inflammatory models in which PPARs were implicated (Table 2) We limited our discussion to those models where molecular details of the regulatory mechanisms are accumulating.

## 5 PPARγ

There are two distinct isoforms of PPARγ, termed PPARγ1 and PPARγ2, which are transcribed from the same gene. PPARγ2 differs by an extra N terminal motif (28 amino acids in human or 30 in mouse) [55]. PPARγ2, which has a stronger transcriptional activity, is expressed at a high level almost exclusively in the adipose tissue, while PPARγ1 is expressed in a host of cell types (including adipose tissue, spleen, liver, pancreas, retina, skeletal muscle, endothelia, vascular smooth muscle cells, sebocytes/sebaceous glands, macrophages, dendritic cells and lymphocytes [25,56–64]) at a lower expression level. PPARγ is an important regulator of adipose tissue development, fatty acid synthesis and insulin sensitivity of major glucose utilizing tissues. PPARγ is required for adipocyte differentiation and for the maintenance of differentiated adipocytes [65,66]. Not only was PPARγ found to be an important regulator of adipose function, lipogenesis and lipid storage, it was also demonstrated that the TZD class of drugs, which can be used for the treatment of type 2 diabetes, are cognate ligands of PPARγ. For this reason PPARγ has received far the most attention among the PPARs. Concomitantly to the studies on the role of PPARγ in metabolism, it was soon realized that PPARγ was not only expressed in adipocytes, but (although at much lower levels) in immune cells, as well. The fact that PPARγ1 was shown to be expressed in inflammatory cells and that there was a group of available synthetic agonists of PPARγ that were approved as drugs (for the treatment of type 2 diabetes) raised the appealing prospect that the same drugs could easily be used to modulate inflammation as well.

### 5.1 Mechanisms of PPARγ mediated gene regulation

The canonical PPAR activity is the ligand dependent transactivation, in which liganded PPARγ forms heterodimers with RXR, and the PPARγ/RXR heterodimers recruit a large protein complex of co-activators required for the regulation of transcription. With the help of these co-activators, PPARγ/RXR heterodimers bound to PPREs in the enhancers of target genes will modulate the activity of the basal transcription machinery. This classic transactivation mechanism is well characterized at the molecular level. It is believed that such ligand mediated PPARγ transactivation usually results in the upregulation and not repression of the target genes.

There are alternative mechanisms by which PPARγ can regulate gene expression. These alternative mechanisms are especially important for the understanding of the regulation of immune response by PPARγ. The reason behind this is that it is very difficult to interpret the role of PPARγ in the immune system solely based on the classic transactivation model. No canonical inflammatory regulators have been described so far that are robustly upregulated by agonist activated PPARγ. Moreover, at least a subset of the anti-inflammatory effects of PPARγ is due to ligand dependent gene repression, which is difficult to reconcile with the classic model of the agonist action of positive regulation of gene expression.

There are a number of distinct mechanisms responsible for ligand-dependent gene repression by PPARγ, all occur through the indirect regulatory effects of ligand binding. The resulting negative regulation of gene repression is termed *trans*-repression if PPARγ action is not mediated by its binding to canonical PPRE target DNA sequences. Maybe the best characterized model is the case of ligand dependent transrepression of inflammatory genes. According to this model [67], there is an inhibitory protein complex bound to the promoter of inflammatory genes (*e.g.* NOS2 (inducible nitric oxide synthase 2)) that keeps these genes repressed in the absence of inflammatory signals. For an efficient expression of NOS2 upon LPS (lipopolysaccharide) induced TLR4 (toll-like receptor 4) activation, two requirements must be met. First, the inhibitory complex must be removed from the promoter, and only then can the key immune regulator NF-κB (nuclear factor kappa-light chain-enhancer of activated B cells) activate NOS2 expression. The removal of the inhibitory complex is normally carried out by the ubiquitin proteasome system. In cells that receive concomitant PPARγ ligand and LPS treatments, a fraction of the liganded PPARγ will be SUMOylated on lysine K365. The SUMOylated PPARγ will not be able to bind its regular heterodimerization partner, RXR. Instead, it will bind to the repressor complex located on the promoter of inflammatory genes. The binding of PPARγ to these repressor complexes will block the ubiquitination and hence the efficient removal of the repressors. As a result, ligand bound PPARγ will maintain the repression on the promoter of inflammatory genes, such as iNOS2, even in the presence of active TLR4 signaling. Other forms of transrepression also exist. PPARγ was shown to bind directly other transcription factors, such as NF-κB or activator protein 1 (AP-1) [68,69], interfering with the DNA binding capacity of these transactivators. Ligand activated PPARγ was also demonstrated to modulate p38 mitogen activated protein (MAP) kinase activity [70]. Although the above mechanisms can explain a subset of the anti-inflammatory effects of PPARγ, it cannot be excluded that positive transcriptional regulation of inhibitory proteins, rather than *trans*-repression of other transcription factors, play important roles in ligand-induced repression. Furthermore, the *in vivo* relevance and the contribution of these proposed mechanisms to the inhibition of gene expression remains to be further established.

### 5.2 Alternative activation of PPARγ

The consequence of ligand mediated activation of PPARγ can be modulated by phosphorylation. The first study described the phosphorylation of PPARγ2 at serine-112 by MAP kinases. This phosphorylation, which reduced the transcriptional activity of PPARγ [71], was observed upon exposure of cells to serum. Controversially, it was also found that insulin potentiated the ligand dependent activation of PPARγ *via* the action of MAP kinases, as witnessed by the enhanced expression of the robust PPARγ target gene, aP2 (also known as FABP4) [72]. PPARγ1 could also be phosphorylated at serine-82 (which corresponds to serine-112 of PPARγ2) by epidermal growth factor (EGF) and platelet derived growth factor [73]. Again, this phosphorylation event attenuated the transcriptional activity of the ligand bound PPARγ. The explanation for the observed divergent effect of PPARγ phosphorylation at the relevant serine is lacking.

Recently a novel mechanism for the posttranslational modification of PPARγ was described [74]. In this study the cdk5 mediated phosphorylation at serine-273 of PPARγ2 led to the dysregulation of the expression of a number of metabolism related target genes in adipocytes. Treatment of adipocytes with the synthetic PPARγ agonist, rosiglitazone, hindered this phosphorylation and normalized gene expression. Importantly, the genes whose expression was perturbed upon serine-273 phosphorylation were known to be relevant in metabolic regulation but did not necessarily belong to the “canonical” genes that are robustly regulated by PPARγ agonists. This is a completely novel angle of PPARγ biology, since until now the synthetic PPARγ ligands (both in metabolism and in other fields, including inflammation) were assumed to work according to either the classical agonist or the transactivation models. In the new model, agonist binding caused a conformational change in PPARγ, which modified the activity of the receptor by blocking a distinct signaling event. Importantly, two different synthetic ligands (rosiglitazone and MRL24), with greatly different agonist activities, were used in the study. In classical agonist studies that measure the expression of target genes upon ligand treatment in cells, MRL24 was found to be a weak agonist of PPARγ, compared to rosiglitazone. Yet, the two ligands inhibited PPARγ serine-273 phosphorylation with similar efficiency. This study raised several important questions. One question is if the same type of serine-273 phosphorylation is important in other functions of PPARγ, *e.g.* in the regulation of inflammation. This is possible because cdk5 was reported to modify the antiproliferative effect of PPARγ [75] and also because cdk5 was shown to be activated in adipocytes exposed to inflammatory signals. The other question is how the gene expression changes caused by the transactivation effect of synthetic ligands relate to the gene expression changes influenced by the cdk5 mediated phosphorylation. The functions of PPARγ in different model systems are usually interpreted based on the agonist action of synthetic ligands. It is possible that the gene expression profile of agonist treated cells does not reflect all the important mode of actions of PPARγ because a different set of genes regulated by a distinct receptor activity remains undetected in these experiments. The finding that weak agonists can work as potent regulators of the cdk5 phosphorylation can also reignite the efforts to find the true endogenous ligands of PPARγ. It is possible that there are molecules that are weak agonists of PPARγ (and therefore were dismissed as endogenous ligands) but are potent regulators of the cdk5 mediated phosphorylation.

### 5.3 Cellular model systems to study the inflammatory functions of PPARγ

Myeloid cells were the first immune cells in which the expression and the function of PPARγ were studied. Murine bone marrow derived and peritoneal macrophages, as well as human monocytic cells (U937) were included in these analyses. Bone marrow macrophages expressed PPARγ at very low levels, but thioglycolate elicited peritoneal macrophages showed a marked upregulation of PPARγ. Additionally, IFN-γ (interferon-gamma) activated macrophages that also received PPARγ ligand treatment phenotypically resembled resting macrophages. The idea was tested if PPARγ is a negative regulator of macrophage differentiation and activation. It was found that treatment of IFN-γ activated macrophages with the synthetic ligand rosiglitazone or with the endogenous ligand 15d-PGJ2 blunted the expression of inflammatory genes, such as the inducible nitric oxide synthase, gelatinase B and scavenger receptor A genes. Interestingly, a number of target sites for immunologically relevant transcription factors (AP-1, NF-kB and STAT1 (signal transducer and activator of transcription-1)) were detected in the proximal and distal promoters of the regulated genes. This suggested that liganded PPARγ acted *via* transrepression of other transcription factors [76]. In a similar study [77], a number of different eicosanoids that are possible endogenous ligands for PPARγ (including 15d-PGJ2), and the TZD drug troglitazone were used to antagonize the effects of different inflammatory signals on human peripheral monocytes. The two unrelated inflammatory agents, LPS and phorbol myristate acetate (PMA), did cause a similar upregulation of inflammatory mediators, such as TNF-α, IL-1β (interleukin-1β) and IL-6 (interleukin-6). Interestingly, however, the putative PPARγ ligand 15d-PGJ2 could block the elevated release of the inflammatory mediators only when PMA was used to activate monocytes. This suggested that PPARγ mediated inhibition of inflammation is specifically restricted to a defined molecular pathway used only by certain inflammatory agents.

In two other early studies human monocytes, the human myelomonocytic cell lines THP1 and HL60 and murine lymph node macrophages [31,58] were investigated. It was found that lipid components of oxidized low density lipoprotein (oxLDL) were able to activate PPARγ in HL60 cells differentiating along the macrophage lineage, while the parent LDL particle did not have the same effect. A screening assay that used the ligand binding domain of PPAR fused to the DNA binding domain of Gal4 identified 9- and 13-HODEs, as the oxLDL derived activators of PPARγ. Because high LDL-cholesterol levels show strong correlation with atherosclerosis, it was proposed the uptake of oxidized LDL by monocytes and the subsequent activation of PPARγ might contribute to the appearance of foam cells, the early hallmarks of atherosclerotic lesions.

The above papers laid the foundation for the field that has seen an ever increasing number of publications on PPARγ functions in myeloid cells since then. Paradoxically, these studies also shed light on the controversies of the field. One such controversy is that 15d-PGJ2, the candidate endogenous ligand used in these and other studies is not a very specific PPARγ ligand. Additionally, the ligand concentration used to inhibit inflammatory responses were higher than it would be expected based on the Kd value for the ligands. For these reasons the involvement of the other PPAR subtypes (or other, unrelated proteins) in mediating the effects of PPARγ ligands could not be excluded. These points are very well illustrated by the findings that showed that 15d-PGJ2 and TZD drugs were similarly effective in inhibiting LPS induced inflammatory cytokine release in wild type as well as in PPARγ^−/−^ macrophages [78]. These results demonstrated that PPARγ ligands (namely TZDs and 15d-PGJ2) could exert potent anti-inflammatory effects *via* PPARγ independent mechanisms. Another caveat in interpreting these and other data is the fact that the observed gene expression changes are not in line with the classical model of positive regulation of target genes by ligand activated PPARγ. Clearly, other models of PPARγ actions, such as ligand dependent or independent *trans*-activation could be responsible for at least a subset of gene expression changes mediated by PPARγ. When the gene expression changes in rosiglitazone treated or untreated murine peritoneal macrophages [79] were characterized, only 8 genes showed modestly enhanced expression when macrophages were treated with high concentration of the synthetic ligand. These genes (including the genes for CD36, adipose differentiation-related protein (ADRP), carnitine palmitoyl transferase 1a (Cpt1a), enoyl coenzyme A hydratase 1 (Ech1) and ATP binding cassette subfamily G1 (ABCG1)) had known functions in lipid metabolism, but did not show evident connection to inflammation. A recent paper [80] demonstrated that activation of PPARγ in murine macrophages regulated the differentiation of the (IL-4 induced) alternative activated macrophages (M2 macrophages). The ability of PPARγ to control the alternative macrophage differentiation was partly mediated by the transactivation of arginase I, a known regulator of the M2 development. The importance of PPARγ in regulating the balance of classical/alternative macrophages in human was also demonstrated by Bouhlel et al. who demonstrated that the level of M2 macrophages in atherosclerotic lesions correlated with the expression level of PPARγ [81]. Interestingly, Szanto et al. suggested that PPARγ is not a regulator of alternative activation of macrophages *per se*, but is more likely a downstream effector of the IL-4 signaling pathway [45]. They found that Il-4 acted as a licensing factor for macrophage PPARγ that promoted PPARγ DNA binding to a subset of PPARγ PPREs in macrophages. Regardless of the signaling hierarchy of IL-4 and PPARγ in M2 macrophage differentiation, PPARγ and IL-4 signaling seem to be mechanistically linked. These reports can redirect the focus of the macrophage PPARγ research from the regulation of classical macrophage activation to other macrophage functions, including alternative macrophage activities. The function of the alternatively activated macrophages is controversial. Due to the fact that alternatively activated macrophages develop in Th2 type immune responses upon macrophage exposure to IL-4 or IL-13, their *in vivo* function is probably linked to IL-4/IL-13 driven processes. These cells have a documented role in immunity against parasites [82,83] and in allergy, and are often implicated in wound healing/repair, angiogenesis and atherosclerosis [84]. The findings of Szanto et al. raise the question of what is the relevance of the PPARγ–STAT6 interaction in lipid metabolism, type-2 diabetes, atherosclerosis and the above, classical IL-4 mediated diseases.

After the characterization of PPARγ in macrophages, a closely related cell type, dendritic cells (DC) came to the spotlight of PPARγ research. Dendritic cells are professional antigen presenting cells that regulate both the innate and adaptive arms of the immune response. Murine immature and mature splenic DCs were shown to express PPARγ [85]. It was also shown that PPARγ was expressed in cytokine treated human monocyte derived DCs (moDCs) [86–88]. Our laboratory and others demonstrated that activation of PPARγ in monocyte derived DCs led to an altered immune phenotype characterized by increased phagocytic capacity, antigen processing and lipid antigen presenting capacity [89–91] and [54]. Although the full picture of how PPARγ activation led to these phenotypic changes in DCs is not fully understood, important regulatory mechanisms were described. In this model system, monocytes were isolated from peripheral blood, and cultured in the presence of GMCSF and IL-4. This led to the differentiation of monocytes into functional moDCs at day 5. It was found that PPARγ expression was transiently induced early in the differentiation pathway. When differentiating moDCs were treated with the synthetic agonist, rosiglitazone, a limited gene expression change occurred within a few hours. The rapid change in gene expression indicated that these genes were most probably directly regulated by ligand activated PPARγ. Several of these directly regulated genes were found to be PPARγ dependent in other cell types (such as FABP4/aP2, angiopoietin-related protein 4 (Angptl4), adipose differentiation related protein (ADRP), CD36). Importantly, several genes responsible for lipid transport/metabolism were induced, but no key regulators of inflammation were found among the acutely regulated genes. When transcriptional changes were monitored for a longer period of time (1 day and 5 days), a new, much more robust set of transcripts (about 1000 transcripts) showed altered expression levels. It is probable that the limited set of directly regulated genes initiated and regulated a secondary wave of transcriptional change, which culminated in the observed phenotypic modulation of moDCs. Such a secondary gene regulation mechanism was identified in our laboratory. PPARγ activation in moDCs turned on metabolic processes that resulted in an enhanced production of retinoic acid, a known regulator of myeloid cell differentiation and immune function. Retinoic acid could activate its own receptors, retinoic acid receptors (RARs), that are also members of the nuclear receptor superfamily. In short, a concerted transition from an active PPARγ signaling to an active RAR signaling occurred [54]. A characteristic phenotypic shift also occurred in PPARγ activated moDCs. These cells had an enhanced capacity to present lipid antigens to invariant natural killer T cells (iNKT cells). This was possible because PPARγ agonist treatment of DCs enhanced the cell surface expression of CD1d molecules by which lipid antigens are normally presented to iNKT cells. An increased lipid antigen presentation by moDCs resulted in the activation and proliferation of iNKT cells.

There are several questions that are raised by the results from studying PPARγ in myeloid cells, including macrophages and DCs. One interesting observation is that the level of PPARγ expression and the activity of PPARγ, even when synthetic ligands are present, do not show strong correlation. Murine peritoneal macrophages express relatively high level of PPARγ, yet they were shown to be rather unresponsive to ligand treatment. There must be other mechanisms, such as the availability of other regulators, co-factors or signaling molecules, that can regulate the responsiveness of PPARγ. This phenomenon might be linked to the fact that a robust PPARγ response can be seen in these cells if they also receive simultaneous IL-4 treatment, which was thought to be mediated by an enhanced PPARγ expression in the presence of IL-4 [92]. An alternative explanation suggested that IL-4 treatment led to an increased production of endogenous ligands in these cells [93]. Recent results, however, raised the possibility that IL-4 enhanced PPARγ response in these cells *via* a third mechanism. IL-4 signaling in these cells is transduced predominantly by the JAK/STAT6 pathway. It was found that upon simultaneous PPARγ and IL-4 activation of macrophages STAT6 was able to bind PPARγ and modified its DNA binding and transcriptional activity [45]. Another interesting feature of PPARγ signaling in myeloid cells is that PPARγ expression and activity are often strongly enhanced transiently during differentiation, *e.g.* during PMA activation of the monocytic THP1 cell line, during the *in vitro* differentiation of human peripheral monocytes into DCs, or during the early stages of murine bone marrow derived macrophage differentiation (unpublished observation). The above features of PPARγ activity in myeloid cells raise the interesting question of which are the physiologically relevant PPARγ responsive cells *in vivo*. It is possible that the typical model cell types that are used to study macrophage/DC PPARγ activity; bone marrow derived macrophages, peritoneal macrophages and monocyte derived macrophages/DCs (all of which require IL-4 signaling for an enhanced PPARγ response) do not represent well the true PPARγ responsive cell populations.

Other immune cells such as T cells were shown to express PPARγ1 [94–96]. It was found that 15d-PGJ2 or TZD (ciglitazone) treatment of murine T cell clones and enriched splenocytes inhibited antigen or anti-CD3 ligation mediated T cell responses. Similarly, Yang et al. reported that human peripheral T lymphocytes produced less IL-2 and showed decreased proliferation upon (PHA) activation in the presence of PPARγ agonist 15d-PGJ2 or troglitazone [97]. An interesting interaction between macrophages and T lymphocytes was also observed. It had been shown earlier [93] that IL-4 treatment of macrophages induced 12/15-lipoxigenase expression in macrophages, which could produce potential endogenous ligands for PPARγ in the essential fatty acid metabolism pathway. Yang et al. demonstrated that T cells cultured in a conditioned medium derived from IL-4 treated macrophages produced significantly less IL-2 upon anti-CD3 ligation or PHA treatment. This result suggested that the PPARγ mediated decrease in the IL-2 production of T cells derived from a non-cell autonomous mechanism.

PPARγ expression was also detected in B cells [98]. B cell response to a various stimuli, including LPS stimulation or antigen receptor crosslinking, was also found to be modulated by PPARγ [99]. Interestingly, PPARγ^+/−^ animals which showed no difference in the T cell compartment when compared to wild type mice exhibited enhanced B cell proliferative responses to stimulation. Contrary to earlier findings, B cells of PPARγ^+/−^ animals exhibited increased viability.

PPARγ expression was also described in isolated primary natural killer (NK) cells and in NK cell lines [100]. Treatment of NK cells with 15d-PGJ2 or ciglitazone was found to cause a general attenuation of NK cell functions. The IFN-γ production, CD69 expression and the cytolytic activity of NK cells were investigated in PPARγ ligand treated (15d-PGJ2 and ciglitazone) and untreated cells. The inhibition of IFN-γ production was mediated by a PPARγ, while the cytolytic activity of NK cells was inhibited by a PPARγ independent mechanism.

### 5.4 PPARγ in animal models of inflammatory diseases

Studying PPARγ activation in isolated primary cells or in cell lines can reveal important regulatory mechanisms by which lipids can influence inflammatory responses of the investigated cell type *via* the modulation of gene expression. It is, however, difficult to predict the *in vivo* consequences of PPARγ activation solely based on the results obtained from *in vitro* studies. First, immune responses can be regulated by many non-cell autonomous mechanisms, and activation of PPARγ in isolated population of target cells fails to detect these regulatory circuits. Second, it is possible that the cell types that can be isolated and cultured in a reasonable quantity do not represent the cell types in which *in vivo* activation of PPARγ has important functions. For these reasons, animal models of disease have been used to elucidate the physiological or pathological roles of PPARγ.

Inflammatory bowel diseases (IBD) (Crohn's disease and ulcerative colitis) are inflammatory conditions in which the role of PPARγ was investigated. There are several murine models for the generation of an intestinal inflammation reminiscent in some aspects to different forms of human IBD. IL-10 knockout mice develop intestinal inflammation spontaneously, while intrarectal administration of dextran sodium sulfate (DSS) or 2,4,6-trinitrobenzene sulfonic acid (TNBS) in wild type mice reliably induces ulcerative colitis. The involvement of PPARγ in inflammatory bowel diseases was investigated in several mouse models and the general picture emerged that PPARγ activity protected from IBD. Due to the fact that PPARγ is normally expressed in both colonic epithelial cells and in resident macrophages of the colonic mucosa, PPARγ activity in both cell types had the potential capacity to ameliorate disease progression. The chemically induced inflammation model was used in mice heterozygous for PPARγ [101], in mice with colonic epithelium specific [102], macrophage specific [103] or T cell specific PPARγ deletion [104], or in wild type mice in which PPARγ ligand treatment was applied to modulate inflammation [105]. All above animal models showed that deficiency in PPARγ resulted in an increased susceptibility to disease. PPARγ deficient animals showed more severe disease symptoms even in the absence of an exogenously administered PPARγ ligand. This suggested that either unliganded PPARγ had an activity that protected mice from the disease, or alternatively, an endogenous ligand with a potent PPARγ agonist activity was produced in these animals. These results raised the possibility that PPARγ agonists could be used to ameliorate human IBD.

Experimental autoimmune encephalomyelitis is an animal model of brain inflammation, in which the role of PPARγ in the regulation of inflammation can be studied. EAE in rodents is accompanied by demyelination. This characteristic of EAE makes it be a useful model for human multiple sclerosis (MS) and acute disseminated encephalomyelitis (ADEM). The first experiments demonstrated that the endogenous ligand 15d-PGJ2 and the TZD drugs troglitazone and ciglitazone were able to ameliorate experimentally induced EAE [106,107]. The decrease in disease severity and duration was, at least partly, due to a decrease in IL-12 production and Th1 cell differentiation. In line with the above results, PPARγ heterozygous mice developed an exacerbated disease [108] in the same EAE model. A recent study suggested that PPARγ activation suppressed central nervous system inflammation in the EAE model *via* the reduction of Th17 T cell differentiation [109]. This study demonstrated both the beneficial effects of the PPARγ agonist treatment in wild type mice and the deleterious effects of the CD4 T cell specific deletion of PPARγ.

Experimental autoimmune myocarditis is another model in which the potential involvement of PPARγ was investigated. When autoimmune myocarditis was induced in Lewis rats by immunization with cardiac myosin, administration of synthetic PPARγ ligands ameliorated disease severity. It was suggested that the inhibition of the expansion of autoreactive T cells and a shift in the Th1/Th2 balance were responsible for the beneficial effects of the agonist treatment [110].

Two medical conditions that develop due to dysregulation of lipid metabolism, atherosclerosis and metabolic syndrome have an underlying chronic inflammatory component. Due to the fact that PPARγ regulates lipid metabolism and also has anti-inflammatory activities, it is a reasonable target in studies that dissect lipid metabolism derived and inflammatory components of these diseases. Li et al. found that rosiglitazone treatment of high fat diet fed animals lead to a reduction of the lesion areas in high fat diet fed LDL-receptor knockout mice. Surprisingly, the beneficial effect of PPARγ activation was detectable only in male mice [111]. The question whether the beneficial effects of PPARγ was mediated by the altered systemic lipid homeostasis or by the altered phenotype of cells involved in the lesions, was investigated by Collins et al. They demonstrated that the anti-atherogenic effects of PPARγ and its beneficial effects on insulin sensitivity were uncoupled, suggesting that PPARγ ameliorated atherosclerosis by affecting cells locally [112]. Additionally, it was shown that bone marrow transplantation of PPARγ^−/−^ cells worsened the outcome of atherosclerosis, strongly suggesting a role for PPARγ in macrophages involved in lesion formation [113].

Unexpectedly, macrophage PPARγ was shown to be a regulator of insulin sensitivity in two metabolic studies [80,114]. It has been known for long from both murine studies and human medical practice that ligand activation of PPARγ improves insulin sensitivity. Originally, it was assumed that the beneficial effects of ligand activation were mediated by PPARγ in one of the major target tissues of glucose and lipid metabolism, such as muscle, fat or liver. Interestingly, the loss of PPARγ in hematopoietic cells was found to lead to insulin resistance. This suggested that inflammation is a component in developing insulin resistance in the mouse and macrophage PPARγ deletion exacerbated this inflammation. It must be added, however, that a similar study [115] found preserved glucose tolerance in high-fat-fed C57BL/6 mice transplanted with PPARγ^−/−^, PPARβ/δ^−/−^ or LXRα^−/−^ (liver X receptor alpha, another nuclear receptor with a role in lipid metabolism) hematopoietic cells. It was also found that the main site of the insulin sensitizing activity of ligand activated PPARγ was, in fact, adipose tissue [116]. Because of these contradicting results further studies are needed to clarify the contribution of macrophage PPARγ to the development of insulin resistance.

### 5.5 PPARγ and inflammation in humans

Human PPARγ mutations and single nucleotide polymorphisms (SNPs) were associated with metabolic and inflammatory diseases. Barroso et al. examined all the coding exons of PPARγ 1 and 2 in 85 unrelated patients with severe insulin resistance [117]. They identified two heterozygous missense mutations of PPARγ ligand-binding domain (P467L and V290M) in three patients. All three patients had type 2 diabetes mellitus and hypertension, suggesting the important role of PPARγ in regulation of insulin sensitivity, glucose homeostasis and blood pressure. Agostini et al. described additional PPARγ mutations found in lipodistrophic insulin resistance patients. These mutations made PPARγ unable to bind DNA and acted in a dominant-negative fashion [118]. Since the canonical function of PPARγ is the regulation of glucose and lipid metabolism, it is more than probable that these phenotypes were caused by the impaired metabolism in these patients and not because the loss of PPARγ contributed to an enhanced inflammation in metabolic disease.

Several single nucleotide polymorphisms (SNPs) of PPARγ were identified. The most commonly studied SNP, Pro12Ala, has firmly been demonstrated to influence metabolic status of patients [119]. Additionally, evidence is accumulating suggesting that PPARγ SNPs also influence inflammatory diseases. Oh et al. showed that the combination of the major allele of the non-synonymous Pro12Ala SNP with the major allele of the synonymous His449His polymorphism was associated with the development of asthma, while the frequency of the major Pro12Ala allele in combination with the minor His449His allele was significantly lower in patients with asthma compared to healthy controls [120]. Penyige et al. showed that major allele of the His447His polymorphisms had protective role in COPD while the minor His447His allele was associated with development of COPD [121]. Additionally, association of the minor allele of the same SNP (referred to as C161T in the study) with ulcerative colitis in the central Chinese but not Dutch IBD population was found [122]. In summary, genetic variations in PPARγ, which have only a slight effect on the function/expression level of the receptor, can have statistically demonstrated effect on several chronic inflammatory conditions. Similar studies in other inflammatory diseases are expected to emerge in the near future and they hold the potential to corroborate or refute models of PPARγ functions that were formulated based on cellular or animal studies.

## 6 PPARα

PPARα expression was predominantly found in the liver, but was also found to be expressed in cardiac myocytes, proximal tubular epithelial cells of kidney, skeletal muscle, large intestine epithelium, endothelial and smooth muscle cells as well as immune cells including macrophages, lymphocytes and granulocytes [5,123–126]. It is a key regulator of peroxisomal and mitochondrial β-oxidation of fatty acids, ketone body synthesis and systemic lipid metabolism. Similarly to PPARγ, there is an accumulating body of data suggesting that PPARα is not exclusively a metabolic regulator, but also have potent anti-inflammatory activities.

### 6.1 Mechanisms of PPARα activation

It is important to note that PPARα is the only subtype of PPARs whose candidate endogenous ligands are indeed most likely *bona fide* ligands. As already mentioned, dietary fatty acids can bind to and activate PPARα. Consequently, there is an intriguing possibility that our diet directly influences our immune system by activating transcription factors and therefore regulating gene expression. The classical model described in the PPARγ section for agonist activated PPAR transactivation is also applicable to PPARα. Similarly to PPARγ, there is hardly any immunologically relevant gene whose positive regulation by PPARα can be explained by the classical, agonist mediated transactivation model. The only notable exception may be the case of IL-4 regulation in lymphocytes [127]. In this experiment, gemfibrozil treatment attenuated the symptoms of experimental autoimmune encephalomyelitis (EAE) in mice. It was observed that the beneficial effect of the gemfibrozil could be detected only in wild type but not in IL-4 knockout animals. Because it was known that gemfibrozil treatment enhanced IL-4 production, the idea was tested if gemfibrozil was a direct regulator of IL-4 expression. Chromatin immunoprecipitation (ChIP) experiments detected PPARα binding to the identified PPRE element in the promoter region of IL-4 and IL-5 genes.

Similarly to PPARγ, PPARα can also regulate gene expression by a variety of transrepression mechanisms. Ligand dependent transrepression was demonstrated in aortic smooth muscle cells [68], where treatment with LPS normally results in IL-6 release. Treatment of aortic explants with fenofibrate strongly decreased IL-6 production. It was found that liganded PPARα directly bound key transcription factors that are known to regulate IL-6 expression, such as the NF-κB subunit p65, c-Jun and c-AMP response element binding protein-binding protein (CBP).

Interestingly, a new twist in the mode of transrepression was described by Bougarne et al. [128]. Glucocorticoid receptor alpha (GRα) is yet another nuclear receptor that has a well documented anti-inflammatory activity. Its ligands, glucocorticoids, are important drugs in treating inflammatory conditions. Both GRα and PPARα can inhibit NF-κB mediated inflammatory gene expression by transrepressing NF-κB. It was found that cells that received simultaneous PPARα and GRα ligand treatments exhibited an increased, additive transrepression of NF-κB. At the same time, ligand activated PPARα could negatively interfere with the transactivation capacity of GRα on its target sequences. These results raised the questions if PPARα, activated by endogenous ligands, is also able to potentiate GRα *in vivo*, and if PPARα agonists could be used in combination therapies with glucocorticoids to alleviate inflammatory conditions.

### 6.2 Alternative activation of PPARα

Several posttranslational modifications were described for PPARα. Insulin mediates the phosphorylation of Ser12 and Ser21, which enhance the transactivation capacity of PPARα [129]. Other phosphorylation mechanisms, by the p38 MAPK or protein kinase C (PKC) pathways, were also described. Interestingly, inhibition of PKC had a dual effect on PPARα. PKC inhibition decreased transactivation capacity of PPARα, but enhanced its transrepression activity [130].

### 6.3 Cellular model systems to study the inflammatory functions of PPARα

The expression of PPARα has been reported in several immunologically relevant cell types. In human monocyte derived macrophages it was found that ligand activation of PPARα induced apoptosis [126]. This pro-apoptotic effect was even more pronounced if the cells were pretreated with INF-γ or TNFα. Jones et al. demonstrated that PPARα was also expressed in T and B lymphocytes, and the expression of PPARα declined when lymphocytes were activated [125]. The ligand activation of PPARα in these cells led to a measurable transactivation activity and the transrepression of NF-κB. Ligand independent transrepression activity of PPARα was found in CD4+ T cells, where unliganded PPARα suppressed the phosphorylation of p38 MAP kinase. PPARα ligand treatment of CD4+ cells led to the relaxation of this suppression effect, and increased p38 MAP kinase phosphorylation [131]. As mentioned above, ligand activation of PPARα in murine T lymphocytes revealed that IL-4 and IL-5 are possible PPARα target genes [127]. Langerhans cells were also shown to express PPARα and pharmacological activation of PPARα inhibited Langerhans cell maturation.

### 6.4 PPARα in animal models of inflammatory diseases

The first report that suggested that PPARα could control inflammation [9] studied leukotriene B4 (LTB4) induced inflammation in wild type and PPARα knockout mice. LTB4 is a locally generated lipid inflammatory agent that initiates and coordinates inflammation by activating its cell surface receptor. Alternatively, it can also bind to and activate PPARα. The activation of PPARα modulates the metabolism of arachidonic acid and its derivative molecules, including LTB4 itself. The idea was tested if PPARα, activated by LTB4, could negatively regulate inflammation by the enhanced metabolism of arachidonic acid (and LTB4), which would result in a limited duration of inflammatory responses. The mouse ear-swelling test (MEST) was used in which the inflammatory agents were locally applied to the ear and ear thickness was measured for the quantitation of inflammatory response. PPARα^−/−^ mice showed prolonged inflammation when arachidonic acid or its metabolite LTB4 were used to trigger inflammation, but the same effect did not occur when a distinct inflammatory agent, phorbol esther was used. Due to the fact that the LTB4 is not only an inflammatory molecule but also an efficient ligand for PPARα, the results suggested the existence of a negative feedback mechanism limiting the duration inflammation.

Interestingly, PPARα showed a dual role in the LPS induced endotoxic shock model [132]. While synthetic agonist treatment of isolated macrophages decreased LPS induced TNFα release, whole body treatment with fenofibrate in LPS treated mice increased the systemic level of TNFα. The proposed explanation for this contradiction was that systemic PPARα activation altered whole body lipid homeostasis, which resulted in an increased propensity to develop endotoxic shock. This could not be offset by a PPARα mediated repression of TNFα in macrophages.

Other animal experimental models also suggested that PPARα has an anti-inflammatory role. In a murine model of human asthma [133] mice were sensitized and challenged with aerosol nebulization of ovalbumin derived antigen (OVA). PPARα deficient mice showed increased eosinophilia, airway hyperresponsiveness, increased IL-6, IL-13 and eotaxin production in lung extracts as well as enhanced serum concentration of antigen-specific IgE. In addition, PPARα activation inhibited IL-5 and eotaxin induced migration and antibody dependent cellular cytotoxicity of human eosinophils *in vitro*.

Delayre-Orthez et al. examined the role of PPARα in another airway inflammation model [134]. Airway inflammation was induced in wild type and PPARα knockout mice by intranasal instillation of LPS. PPARα deficient mice exhibited increased number of neutrophils and macrophages and released higher level of TNFa, MIP-2 (macrophage inflammatory protein-2), MCP1 (monocyte chemoattractant protein 1) and KC/CXCL1 (keratinocyte chemoattractant) in the bronchoalveolar lavage fluid. In addition, the PPARα ligand fenofibrate inhibited LPS induced macrophage and neutrophil infiltration, decreased cytokines levels and MMP-2 and MMP-9 (matrix metalloproteinase) activity in wild type but not knockout mice. This demonstrated that the effects of fenofibrate were specifically mediated by PPARα.

In the carrageenan-induced paw edema and carrageenan-induced pleurisy models of inflammation, Cuzzocrea et al. found that deletion of PPARα led to an increased paw edema and pleural exudate formation and neutrophil infiltration [135]. Furthermore, PPARα^−/−^ animals exhibited enhanced production of TNFα, IL-1β in the pleural exudate. Interestingly, D'Agostino et al. demonstrated that the acute intracerebroventricular administration of an endogenous PPARα ligand, palmitoylethanolamide, had potent anti-inflammatory effects in the carrageenan-induced paw edema model when palmitoylethanolamide was administered prior to the carrageenan injection [136]. Importantly, intracerebroventricular administered palmitoylethanolamide failed to modulate inflammation in PPARα knockout mice. Again, this proved that the anti-inflammatory effects of palmitoylethanolamide were mediated by PPARα.

Several experiments suggest a potential role of PPARα in colonic inflammation and maintenance of mucosal tissue homeostasis. In the IL-10 knockout mice model, Lee et al. demonstrated that the PPARα ligand fenofibrate could decrease colonic INF-γ and IL-17 expression and leukocyte infiltration [137]. PPARα was expressed in lymphocytes, macrophages and colon epithelial cells in this model. Therefore it was not determined in which cell type PPARα activation was required for the amelioration of the disease. Similarly, in the DSS induced colitis model, administration of another synthetic PPARα ligand, WY-14643, resulted in a reduced release of inflammatory cytokines (including INF-γ, IL-1β, IL-6 and TNFα) [138].

Other studies investigated the role of PPARα in mouse model of atopic dermatitis. Atopic dermatitis can develop in mice following topical sensitization and challenge with ovalbumin or oxazolone. Staumont-Sallé et al. described that PPARα deficient mice showed enhanced dermal recruitment of inflammatory cells, IgG2a and IgE production, epidermal thickening as well as lung inflammation and airway hyperresponsiveness following sensitization with ovalbumin [139]. In addition, topical application of the PPARα ligand, WY-14643, significantly decreased antigen-induced skin inflammation in the ovalbumin induced atopic dermatitis model. In the oxazolone induced atopic dermatitis model, the topical activation of PPARα by WY-14643 could normalize the structure of epidermis, significantly improved barrier function of the skin and increased stratum corneum hydration as well as reversed the immunologic abnormalities. Furthermore, PPARα activation decreased epidermal production of inflammatory cytokines in both ovalbumin and oxazolone induced atopic dermatitis [140].

The role of PPARα in experimental autoimmune encephalomyelitis was also investigated.

Lovett-Racke et al. demonstrated that PPARα agonists could be used to ameliorate disease progression [141]. The oral administration of gemfibrozil and fenofibrate inhibited clinical signs of experimental autoimmune encephalomyelitis and also suppressed antigen specific T cell proliferation in a dose dependent manner. The PPARα agonists also increased the production of the Th2 cytokine IL-4 and inhibited the release of the Th1 specific IFN-γ. The agonists also decreased nitric oxide (NO) production by murine microglial cells and enhanced IL-4 production and suppressed IFN-γ secretion of cultured human T cells. It must be noted, however, that Dasgupta et al. reported that the gemfibrozil induced reduction of clinical signs of EAE, demyelination and infiltration of mononuclear cells in the central nervous system, were independent of PPARα [142]. In a third study, male but not female mice exhibited enhanced clinical signs of inflammation and increased numbers of inflammatory brain lesions in PPARα^−/−^ animals. PPARα expression in CD4+ T cells was found to be higher in male than in female mice, suggesting sex specific role of PPARα in T cells mediated autoimmunity [143,144]. In addition, Xu et al. demonstrated the effect of activated PPARα on primary mouse astrocytes, a cell type implicated in the pathology of MS and EAE. Fenofibrate and WY-14643 were able to inhibit the NO production of astrocytes stimulated by LPS in a dose-dependent manner. Furthermore, fenofibrate treatment of astrocytes decreased the LPS induced secretion of TNFα, IL-1β and IL-6 [145].

The liver is the centre of the lipid and carbohydrate metabolism as well an important regulator of inflammation. Acute phase proteins are among the key molecules by which liver is able to modulate inflammation are. PPARα was demonstrated to influence systemic inflammatory responses by regulation of the acute phase response. The PPARα ligand fenofibrate inhibited the expression of IL-6 induced acute phase response proteins including fibrinogen-a, -b and -g, serum amyloid A and haptoglobin in the liver of wild type but not in PPARα knockout mice. As an additional proof for the role of PPARα in acute phase response, fenofibrate treated hyperlipidemic patients were shown to have decreased plasma concentration of fibrinogen, C-reactive protein, serum amyloid A, plasminogen and a2 macroglobulin [146].

PPARα has the potential to affect atherosclerosis at two levels. First, PPARα is a regulator of lipid metabolism, and the loss of PPARα function causes higher than normal levels of atherogenic lipoproteins. Alternatively, PPARα could impact the inflammatory component of atherosclerosis. Tordjman et al. demonstrated that PPARα/ApoE double knockout mice showed higher serum level of atherogenic lipoproteins, but decreased aortic atherosclerotic lesions, lowered insulin resistance and blood pressure as compared to PPARα^+/+^ ApoE^−/−^ littermates [147]. Interestingly, Babaev et al. examined the role of PPARα in bone marrow-derived cells in atherosclerosis by reconstituting LDLR knockout mouse hematopoietic tissue with bone marrow from PPARα knockout and wild type mice. LDLR^−/−^ mice following bone marrow transplantation from PPARα^−/−^ mice showed increased size of atherosclerotic lesions in aorta compared to LDLR^−/−^ mice with wild type derived bone marrow, suggesting complex role of PPARα in development of atherosclerosis [148].

Similarly to PPARγ, it is possible that PPARα activity also modulates obesity associated inflammation either through its metabolic activity or anti-inflammatory effects. Induction of obesity with high-fat diet in PPARα knockout or wild type mice suggested that PPARα protected against obesity-induced chronic inflammation in the liver. Plasma markers of liver injury and inflammation, including serum amyloid A and alanine aminotransferase activity, were increased in high-fat diet fed PPARα^−/−^, but not in wild-type animals [149].

## 7 PPARβ/δ

Among the PPARs, PPARβ/δ has been the least studied subtype so far. However, important advances were made in recent years in understanding the metabolic and inflammatory functions of PPARβ/δ. PPARβ/δ is expressed almost ubiquitously [59], with the highest level of expression found in colon, small intestine, liver and keratinocytes. PPARβ/δ is a general regulator of fatty acid oxidation in many tissues.

### 7.1 The role of PPARβ/δ

The involvement of PPARβ/δ in the regulation of lipid metabolism has been well established based on knockout and overexpression studies in transgenic mice [49,150,151]. PPARβ/δ knockout mice are smaller, both pre-, and postnatally, than wild type animals. Reduced offspring numbers were also found, due to a placental defect. Overexpression of a constitutively active PPARβ/δ in white adipose tissue reduced adiposity, most probably to the enhanced level of fatty acid oxidation. The metabolic pathways that were under the regulation of PPARβ/δ included fatty acid metabolism, mitochondrial respiration and programming of the muscle fiber type. The analysis of the full body knockout of PPARβ/δ revealed that it also regulated the inflammatory reaction during skin wound healing [150] and [151]. Recently, several studies reported the role of PPARβ/δ in different types of inflammation.

### 7.2 Cellular model systems to study the inflammatory functions of PPARβ/δ

Several inflammatory cell types express PPARβ/δ. PPARβ/δ activity in macrophages was thoroughly studied due to the connection of PPARβ/δ to atherosclerosis. Several inflammatory genes are regulated by PPARβ/δ in murine macrophages, *e.g.* monocyte chemoattractant protein 5 (MCP-5/CCL12), IL-1β, TNFα, IL-6 and VCAM-1 (vascular cell adhesion molecule 1) [152,153]. The main form of PPARβ/δ activity in these cells is proposed to be a ligand-independent transrepression, in which unliganded PPARβ/δ binds and sequesters the repressor molecule B-cell lymphoma-6 (BCL-6). Upon PPARβ/δ ligand activation, however, PPARβ/δ releases BCL-6, which then can repress the expression of inflammatory genes.

### 7.3 PPARβ/δ in animal models of inflammatory diseases

Skin inflammation and wound healing was the first inflammatory model in which the role of PPARβ/δ was investigated. Michalik et al. found that all PPARs were expressed in the skin during fetal development of mice [151]. Interestingly, although the expression of all PPARs in healthy adult epithelium declined, PPARα and PPARβ/δ were rapidly reactivated in adult epidermis upon a variety of inflammatory stimuli, including local tetradecanoyl phorbol acetate (TPA) application on skin and skin wounding. The two PPARs affected different stages of wound healing; PPARα was important in the regulation of the early inflammatory reaction, whereas PPARβ/δ regulated keratinocyte proliferation. PPARα^−/−^ animals showed impaired infiltration of immune cells to the site of the injury, which resulted in delayed healing. On the other hand, PPARβ/δ^+/−^ heterozygous animals exhibited a hyperproliferative keratinocyte reaction.

The involvement of PPARβ/δ in atherosclerosis was also investigated [154]. It was found that PPARβ/δ deficiency in hematopoietic cells protected against atherosclerosis. Interestingly, it seems that the loss of PPARβ/δ in foam cells reduced atherosclerotic lesion areas not by the modulation of lipid metabolism but the regulation of the inflammatory component of atherosclerosis. The expression of several inflammatory genes was decreased in PPARβ/δ deficient macrophages, including MMP9 (matrix metalloproteinase 9) and IL-1β. According to the proposed mechanism for the observed anti-inflammatory role of PPARβ/δ, a ligand independent transrepression of BCL-6 was proposed. Also a ligand independent transrepression mechanism was proposed for the protective effect of PPARβ/δ in DSS induced colitis. The level of IFN-γ, TNF-α and IL-6 was increased in PPARβ/δ deficient animals [155].

Interestingly, PPARβ/δ also influenced the development of alternatively activated macrophages (M2 macrophage). Arg1, an M2 marker gene that is under PPARγ regulation in murine myeloid cells [80], was also regulated by PPARβ/δ [156,157]. In fact, a complementary regulation by both PPARγ and PPARβ/δ was needed to ensure Arg1 expression. Moreover, the M1 macrophage mediated uptake of *Leishmania major* was impaired in cells that received PPARβ/δ ligand treatment. These results suggested again that PPARγ and PPARβ/δ, but not PPARα, are regulators of the M2 macrophage development.

Another animal model in which PPARβ/δ shows overlapping functions with other PPARs is EAE [143]. In the EAE model, PPARβ/δ^−/−^ animals displayed more inflammatory foci in the central nervous system. This was partly due to an expanded population of CD4+ cells that produced both IFN-γ and IL-17. PPARβ/δ ligand treatment of wild type CD4+ cells in serum-free medium resulted in a decreased production of IFN-γ by wild type but not PPARβ/δ^−/−^ cells.

Recently, another aspect of the anti-inflammatory role of PPARβ/δ was revealed [158]. PPARβ/δ was found to be necessary for the timely clearance of apoptotic cells. PPARβ/δ deficiency caused a delay in the uptake, while PPARβ/δ ligand treatment in wild-type mice caused an enhanced uptake of apoptotic cells. C1q, a component of the classical complement activation pathway was found to be a direct target gene of PPARβ/δ. As a result of the abnormal sensing of apoptotic cells in PPARβ/δ^−/−^ animals, these mice developed a lupus erythematosus-like autoimmune disease.

## 8 Summary and perspectives

PPARs are a truly fascinating set of molecules. They represent a direct link between changes in the intracellular lipid milieu and the expression of the genome. It is intriguing to see how literature on the activity of these transcription factors is broadening, largely due to the availability of synthetic ligands, animal models and drugs acting *via* these proteins. However, in spite of the wealth of the sometimes conflicting pieces of information available, key issues are still unresolved. These include the true nature of ligand(s) and the way it/they are generated, the predominant mechanisms of action, the genomic loci regulated along with target genes and the tissue and disease specific contribution of the individual receptors to cellular function and diseases. Further progress will be fuelled by emerging novel approaches and methodologies such as *in vivo* ligand detection and determination and single cell biophysical and microscopic technologies to detect ligand–receptor and ligand–co-factor interactions *in vivo*. Systematic efforts to determine genomic binding sites, the identity of co-binding transcription regulators and associated histone marks to reveal the predominant mechanisms of action including the often overlooked transcriptional repression are also needed. Finally the field needs to go down the painstaking path of evaluating the role of each of the three receptors in individual cell types using advanced mouse genetics approaches combined with disease models to truly define the regulated gene sets and pathways. These along with clinical advances can provide us with the insights into the intricate relationship between lipids, receptors and inflammation. Identification of mechanisms of endogenous ligand generation and the direct target genes of each of the receptors in every relevant tissue would be particularly useful in order to identify new therapeutic targets for the biotech and pharma industries. Such new developments would provide further and a much needed boost for the field.

## Figures and Tables

**Table 1:** PPAR ligands. See text for details.

Receptor	Endogenous ligands	Synthetic ligands
PPARα	Saturated fatty acids, unsaturated fatty acids, leukotriene B4, 8-HETE	Clofibrate, fenofibrate, gemfibrozil, Wy-14643
PPARβ/δ	Saturated fatty acids, unsaturated fatty acids, 15-HETE, components of VLDLs	GW-501516
PPARγ	Unsaturated fatty acids, 15d-PGJ2, 15-HETE, 9-HODE, 13-HODE, components of oxLDLs	TZDs (rosiglitazone, pioglitazone, troglitazone and ciglitazone), farglitazar, tyrosine derivatives, NSAIDs

**Table 2:** Mouse inflammatory models and PPARs. Relevant inflammatory models and the involvement of PPAR subtypes are shown.

Experimental model	PPARα	Ref.	PPARβ/δ	Ref.	PPARγ	Ref.
Experimental autoimmune encephalomyelitis	Ligand treatment: ↓severity ↑IL-4, ↓IFN-γ ↓NO production	[141,142,144]	Ligand treatment: ↓severity	[143]	Ligand treatment: ↓severity ↓IL-12, Th1/Th17 T cells	[106,107,109]
			PPARβ/δ^−/−^ mice: ↑severity ↑IFN-γ, IL-12		PPARγ^+/−^ mice: ↑severity	
Colitis	Ligand treatment: ↓severity ↓INF-γ, IL-17	[137,159]	Ligand treatment: No effect	[155]	Ligand treatment: ↓severity	[101,105]
	PPARα^−/−^ mice: ↑severity		PPARβ/δ^−/−^ mice: ↑severity		PPARγ ^+/−^ mice: ↑severity	
Carrageenan-induced	Ligand treatment: ↓severity	[135]	Ligand treatment: ↓severity	[160]	Ligand treatment: ↓severity	[161]
edema (acute inflammation)						
Atherosclerosis	PPARα^−/−^ mice: ↓/↑lesions	[147, 148]	Ligand treatment: ↓lesions	[152]	Ligand treatment: ↓lesions (only in males)	[111,113]
	PPARα^−/−^ BMT: ↑lesions				PPARγ^−/−^ BMT: ↑lesions	
